# Investigating the Structural Basis of Diacetyl Recognition by the G‑Protein-Coupled Receptor ODR-10 in *Caenorhabditis elegans*

**DOI:** 10.1021/acs.jpcb.5c04257

**Published:** 2025-11-14

**Authors:** Lorenzo Di Rienzo, Edoardo Milanetti, Viola Folli, Giancarlo Ruocco

**Affiliations:** † Center for Life Nano & Neuro Science, 121451 Istituto Italiano di Tecnologia, Viale Regina Elena 291, 00161 Rome, Italy; ‡ 528488 UniCamillus-Saint Camillus International University of Health Sciences, Via di Sant’Alessandro 8, Rome I00131, Italy; § Department of Physics, 9311 Sapienza University of Rome, Piazzale Aldo Moro 5, 00185 Rome, Italy

## Abstract

G-protein-coupled receptors (GPCRs) are among the most versatile molecular sensors in biology, capable of sensing and responding to a wide range of molecules and serving as key targets in drug discovery. In *Caenorhabditis elegans*, the GPCR ODR-10 is essential for olfactory detection of diacetyl, a crucial cue for chemotaxis. However, the structural details of this interaction remain poorly understood. In this study, we combined extensive molecular dynamics simulations and docking experiments to gain insight into the structural determinants of diacetyl recognition by ODR-10. Our results revealed that the transmembrane region of ODR-10 is highly stable, allowing us to extract representative conformations for docking studies. Thus, through molecular docking, we identified the protein pocket involved in ligand recognition: the fitness of such a region as a diacetyl binder was further demonstrated by additional molecular dynamics simulations that highlighted the stability of the complex. Finally, we performed a computational alanine scanning mutagenesis procedure over all the binding site residues, demonstrating that specific aromatic and polar residues contribute significantly to binding affinity, and their mutation leads to a substantial loss of interaction. Moreover, turning the attention to the diacetyl conformation, we found that it adopts a very preferred conformation alone in solution but displays a more balanced conformational distribution when bound, suggesting that its conformation is not crucial in receptor binding. This study sheds light, at the atomic scale, on the structure of this key interaction within the olfactory system of *Caenorhabditis elegans*, which is significant both from a theoretical perspective and for potential biotechnological applications.

## Graphical abstract

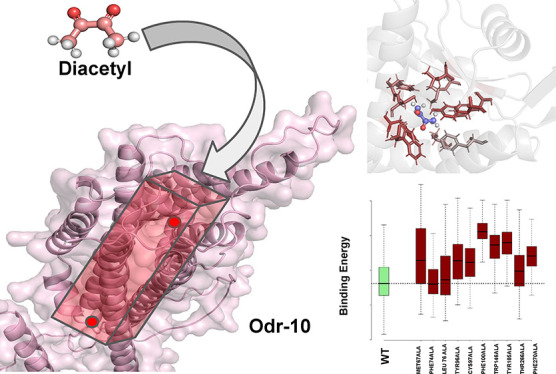

## Introduction

G-protein-coupled receptors (GPCRs) are the largest family of transmembrane proteins, mediating the cellular response to external stimuli, such as light, odors, hormones, and growth factors.^1^ The recognition in the extracellular environment of an agonist ligand drives conformational changes of the protein, which assumes a conformation able to bind the corresponding G-protein in the cytosol. This pairing initiates the cascade of biochemical events, leading to the cellular reaction.^2,3^ From an applicative point of view, GPCRs are among the most pharmaceutically relevant protein families, representing key targets for a wide range of therapeutic drugs.^4^ Moreover, GPCRs also hold significant potential in biosensor development since their design has proven to be effective in the detection of user-defined molecules.^5–7^ In this panorama, the olfactory receptors (ORs) constitute the largest subfamily belonging to class A of GPCRs. They are present in all multicellular organisms and share a common structure formed by seven transmembrane helices.^8,9^

In this context, the nematode *Caenorhabditis elegans* serves as a valuable model for investigating the biology of olfaction. Indeed, it is equipped with 302 neurons, of which only 32 are dedicated to chemosensing.^10^ However, this tiny roundworm can detect a vast array of odors and exhibit many olfactory-driven behaviors.^11–13^

It is important to note here that, in mammals, it is widely accepted—though with some exceptions^14^—that each olfactory neuron expresses only a single type of OR.^15–17^ This strict separation of ORs across different olfactory cells ensures precise odor discrimination as signals from neurons expressing the same receptor converge in the olfactory bulb. Conversely, in C. elegans, each olfactory neuron presents multiple GPCR types on its membrane, allowing it to detect a broader range of odorants compared to mammalian neurons.^18,19^ Furthermore, the same GPCR can be expressed in different neuron types, and a single odorant can bind to multiple receptors located on functionally distinct neurons.^20^ Depending on its concentration, the same odorant may even elicit opposing behavioral responses.^21^ This results in an extremely high level of combinatorial complexity in odorant–receptor interactions within the nematode,^22,23^ and it is not possible to easily derive the receptor–ligand pairing using dose–response curves, as it works in different animals.^19,24^

In this panorama, one of the most characterized OR-ligand pairings in C. elegans regards the interaction between ODR-10, an OR highly expressed on AWA neurons (one of the three core sensory classes of neurons), and diacetyl. Although AWA neurons respond to multiple odorants via ODR-10-independent mechanisms, indicating the existence of additional receptors,^25–27^ and diacetyl can still activate sensory neurons that do not express ODR-10,^18^ this receptor remains the only one currently identified for diacetyl.^28,29^

However, structural information about the molecular complex between these two molecules is still lacking in terms of both ligand recognition and consequent protein activation. Here, we present a computational analysis based on extensive molecular dynamics simulations and molecular docking, investigating the details of diacetyl recognition by ODR-10 at atom-level resolution.

We initially investigated the motion of the protein within the membrane when it was simulated without the ligand. This analysis revealed that the transmembrane region of the protein exhibits overall stability. Alongside, we also investigated the conformational preferences of unbound diacetyl, simulating it in molecular dynamics in solution. We hence performed extensive molecular docking simulations with diacetyl, constraining the system to dock within the transmembrane helical region, usually responsible for agonist ligand binding in class A GPCRs. After selecting two docking poses, we conducted a new simulation of the ODR-10–diacetyl complex, showing that both simulations converged toward a common binding mode, despite their significantly different initial conditions. This allowed us to identify the protein ligand binding mode and ODR-10 residues most involved in diacetyl binding. As further validation, we performed computational alanine scanning, mutating each of these ten residues to alanine and analyzing the system’s behavior in simulation. Both contact analysis and MM/PBSA^30^ interaction energy calculations performed using g_mmpbsa^31^ indicated that these mutations resulted in a weaker binding interaction.

Therefore, we concluded about the reliability of the findings regarding the ODR-10 residues involved in agonist ligand recognition.

## Results and Discussion

### Analysis of the Stability of Unbound Molecular Species

In this section, we first investigated the mobility of the ODR-10 protein in its unbound conformation. To date, the experimental structure of this protein is not yet available in the Protein Data Bank;^32^ therefore, we worked with the structure predicted by AlphaFold,^33^ which is accessible on UniProt^34^ under the entry for this protein. However, given the high degree of structural conservation exhibited by GPCR proteins, the confidence provided by AlphaFold in its prediction is generally high. Usually, the structure of a GPCR provided by AlphaFold corresponds to the receptor in its inactive state. In this work, we investigate the binding between an agonist ligand and its cognate GPCR; therefore, the inactive conformation is precisely the one we intended to use since ligand binding itself is expected to trigger GPCR activation and the associated conformational change. However, it is important to note that in general, no experimental information is available regarding the conformational state of this receptor. We have nonetheless attempted to mitigate this limitation by performing an extensive molecular dynamics simulation, thereby allowing the system to explore its conformational space as broadly as possible.

Therefore, to study the conformations explored by this protein at equilibrium in molecular dynamics, we set up a full-atom simulation in which the protein is embedded in a membrane with explicit solvent (summing up to a total of more than 150k atoms). We thus simulated such a system for 1 μs, and the obtained results are summarized in Figure 1, where it is shown also a snapshot of the complete molecular system under examination.

**Figure 1:**
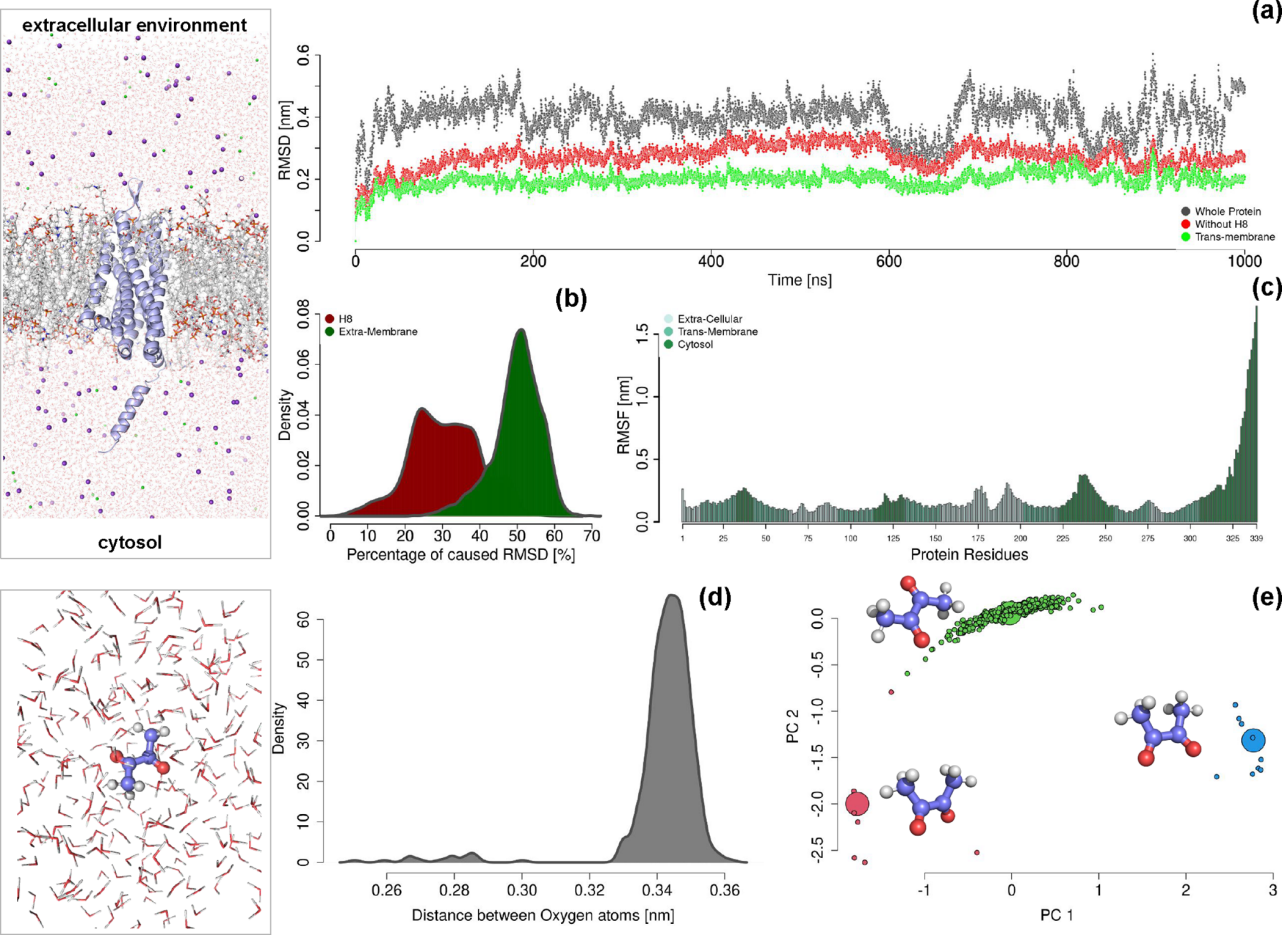
Molecular dynamics simulation of unbound molecular species.(a) RMSD of the protein with respect with the initial configuration, as a function of time. The gray line depicts the time course observed when considering the backbone atoms of the whole protein. The red line describes the RMSD calculated as before but not considering the helix H8. The green line reports the RMSD time course regarding the transmembrane part only. (b) Distribution of RMSD percentages attributed to the H8 helix and the entire nonmembrane region, shown in dark red and dark green, respectively. (c) RMSF of the protein residues. The color code accounts for the domain to which the residues belong: extracellular, transmembrane, or cytoplasmic. (d) Distribution of the distance values observed between the two oxygen atoms of diacetyl in molecular dynamics simulation frames. (e) Principal component analysis on the molecule coordinates: representation of the diacetyl frames in terms of the first two principal components. The colors characterize the three main clusters that are recognizable in the plot. For each cluster, the larger point represents the centroid, whose molecular conformation is also depicted. In the **right** molecular representations, we reported the two simulated systems, ODR-10 embedded in membrane in the **top** panel and diacetyl in solution in the **bottom** panel.

We first analyzed the time course of the root mean square deviation (RMSD) of the protein’s backbone atoms, as shown in Figure 1a. As observed in the gray curve, after an initial equilibration phase, the RMSD remains generally stable. However, particularly in the second half of the simulation, some RMSD discontinuities suggest the occurrence of minor conformational changes. Thus, we investigated which protein regions were responsible for these instabilities, focusing initially on the role of the H8 helix, which is structurally positioned in the cytosol. In the same plot, the red curve represents the RMSD of the protein calculated without considering this helix. As expected, the RMSD value is lower; however, it also shows a significant reduction in instability. This trend is further confirmed and amplified when analyzing only the membrane-embedded portion of ODR-10. Specifically, when considering only the transmembrane region (green curve), the RMSD remains remarkably stable throughout the simulation.

To better quantify this aspect, Figure 1b displays the distributions of the RMSD attributed to these regions. For each frame, we calculated the ratio of the RMSD for the entire protein, excluding the selected region, to the RMSD of the full protein. Reporting 100% minus this ratio, we have a number expressing the importance of such a region in determining the protein RMSD, where a high number accounts for a remarkable importance. Interestingly, on average, helix H8 is responsible for more than 30% of the total RMSD, and, in some frames, it accounts for about 60%. On the same line, on average, the nonmembrane part of ODR-10 causes 49.9% of the RMSD, with peaks reaching up to 70%.

To further analyze residue motion, we studied the root mean square fluctuation (RMSF) of residues with respect to their average position. Looking at the barplot in Figure 1c, it emerges that H8 is actually responsible for most of the fluctuations. In particular, the transmembrane helices of the protein are all characterized by very low RMSF values (cyan bars in Figure 1c).

Taken together, these results suggest that at equilibrium, the most significant protein fluctuations are associated with H8 and the extracellular regions. In contrast, the transmembrane domain, which rules the ligand recognition, remains highly stable. This finding allows us to conclude that the transmembrane region of the tetramer of ODR-10 primarily explores a single macrostate, enabling us to uniformly sample frames for the docking simulations described in the next section.

In parallel, we investigated the possible conformers of diacetyl by analyzing the conformational space explored by this molecule through molecular dynamics simulations. Specifically, we simulated a single copy of diacetyl in solution for 500 ns. The conformations explored by diacetyl can be broadly classified as cis and trans, which differ based on the relative positioning of the two methyl groups with respect to the central C=C double bond. To unambiguously distinguish between these two conformations, we used the distance between the two oxygen atoms in diacetyl as a descriptor since the molecule adopts the cis conformation when the oxygens are close and the trans conformation when they are farther apart. Figure 1d shows the distribution of this distance obtained by analyzing all frames from the molecular dynamics simulation. It is evident that the trans conformation—characterized by an oxygen–oxygen distance around 0.35 nm—is largely predominant and thus energetically favored when the molecule is unbound in solution. In fact, only 3.5% of the frames adopt a cis conformation, using 0.32 nm as the threshold distance.

To further analyze this structural variability, we performed a principal component analysis (PCA) of the atomic coordinates of the small molecule throughout the simulation. Figure 1e shows the scatter plot of the conformations projected onto the principal plane defined by PC1 and PC2, which together account for over 88% of the total variance. Three distinct clusters emerge, shown in different colors in the figure and obtained via k-means clustering. The dominant cluster, shown in green, contains the vast majority of the conformations. Its centroid is also depicted in the figure and corresponds to a trans conformation with the oxygen atoms far apart. The remaining two clusters, which together account for just over 3.5% of the population, both correspond to cis conformations, although they differ in the relative stretching of the oxygen atoms.

Taken together, these results suggest that the distance between the oxygen atoms is a suitable descriptor for distinguishing between trans and cis conformations, although it lacks the sensitivity to capture more subtle differences within the cis ensemble, as revealed by PCA.

### Molecular Docking between ODR-10 and Diacetyl

In the previous section, we demonstrated that the transmembrane portion of the agonist ligand of ODR-10, which is responsible for recognizing the agonist ligand, primarily exists in a single stable state. This observation suggests that the receptor explores a limited conformational space consistent with a single dominant state. For this reason, instead of performing an explicit clustering analysis of receptor conformations, we opted to uniformly sample the ODR-10 structures along the MD trajectory. Specifically, we selected one ODR-10 structure every 20 ns of simulation, disregarding the initial 200 ns equilibration phase. As a result, we obtained 42 independent ODR-10 structures that represent the equilibrium dynamics.

For each of these structures, we performed a molecular docking simulation using AutoDock Vina,^35,36^ restricting ligand binding to occur within the central transmembrane of ODR-10, formed by its helices. Specifically, we calculated the centroid of the extracellular and transmembrane residues of ODR-10, defining a parallelepiped-shaped docking region within which the ligand could bind (see the Materials and Methods section for details). Indeed, for many class A GPCRs, it has been reported that agonist binding often occurs within the transmembrane region, typically involving helices 3, 5, and 6,^37–39^ and this region is fully contained within our defined docking box. Notwithstanding this, we note here that not all of the class A GPCRs share this structural property. Therefore, in this work, we hypothesize the transmembrane region as the most plausible binding site based on structural analogy with other class A GPCRs. The docking box was thus ad hoc defined to encompass this area. Importantly, it should be emphasized that AutoDock Vina allows the ligand to explore multiple conformers during the docking process, the distribution of which will be discussed later in this section.

As a result, we obtained a total of 702 docking poses from molecular docking simulations. These poses were analyzed, and the results are presented in Figure 2. In Figure 2a, we present the distribution of the binding affinities predicted by the docking software. Notably, more than 78% of the poses exhibit a predicted affinity lower than 0 kcal/mol, while approximately 60% show an affinity lower than −2 kcal/mol. We then selected these high-affinity poses (affinities better than −2 kcal/mol) and analyzed the ODR-10 residues consistently involved in interactions with diacetyl.

**Figure 2:**
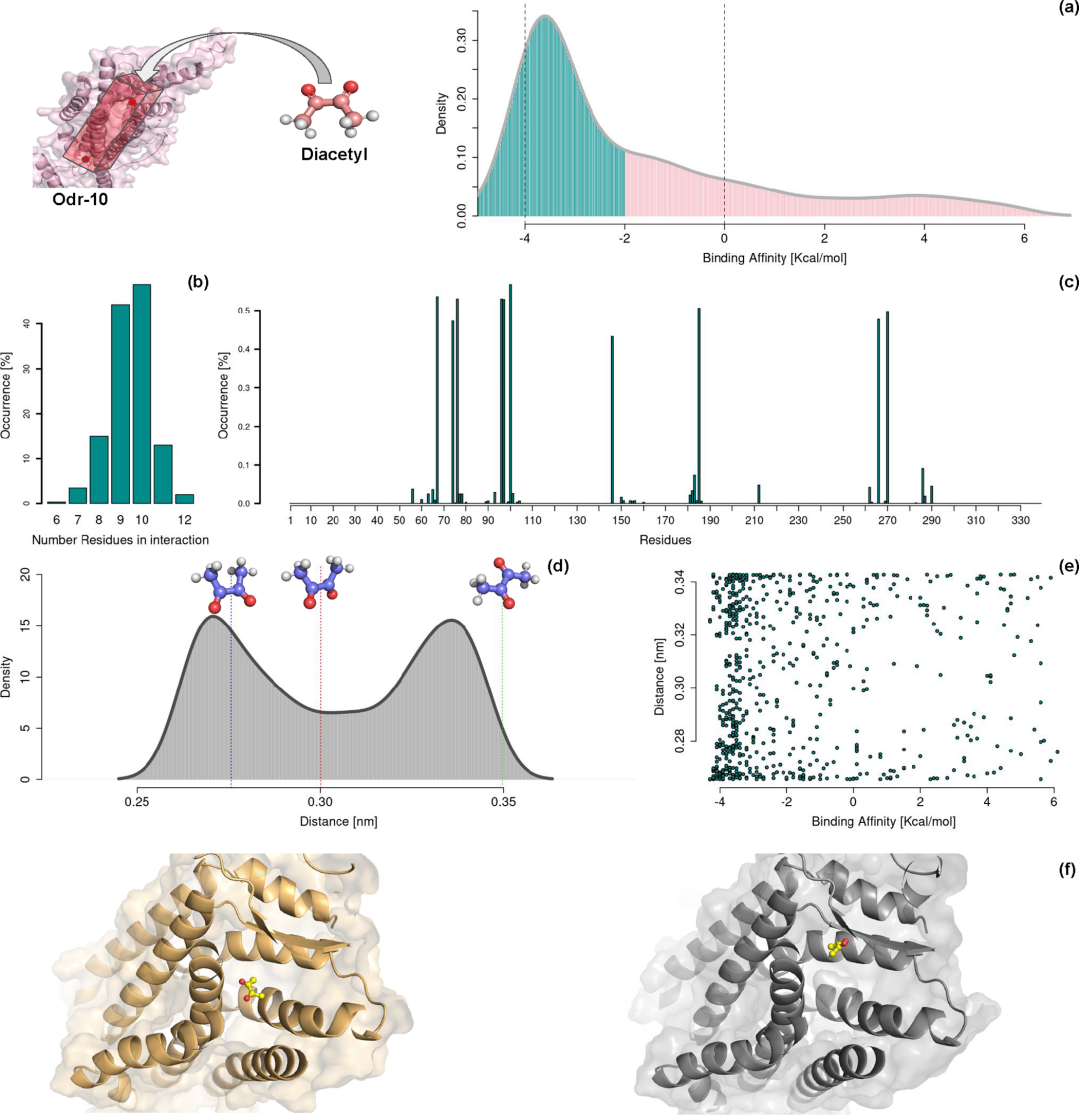
Molecular docking between the ODR-10 protein and diacetyl. In the upper left panel, we reported a schematic illustration of the imposed geometrical constraints. (a) Distribution of the predicted binding affinity values provided by AutoDock software for the 702 obtained docking poses. The curve is colored cyan or pink by using −2 kcal/mol as the threshold value. The vertical dashed lines (−4 and 0 kcal/mol) separate the highly favorable poses and the highly unfavorable pose, from where we identified the two poses for further molecular dynamics validation. (b) Histogram reporting the number of residues each time in interaction with diacetyl, considering only the poses with a predicted binding affinity lower than −2 kcal/mol. (c) Histogram reporting, for each residue, the occurrence of interaction with diacetyl, considering only the poses with a predicted binding affinity lower than −2 kcal/mol. (d) Distribution of the distance values observed between the two oxygen atoms of diacetyl in all of the docking poses. The dashed lines report for the values observed in the three centroids of the clustering procedure performed in the previous section. (e) For each docking pose, we plotted the oxygen distance as a function of the predicted binding affinity. (f) Selected favorable (left) and unfavorable (right) protein–ligand docking poses.

We define a residue as being in contact with the ligand when at least one of its atoms is within 4 Å of molecular partner atoms.^40,41^ We present in Figure 2b a histogram showing the probability of a pose having a given number of interacting residues. It can be observed that the most probable values are 9 or 10 interacting residues. Interestingly, analyzing which residues are most involved in binding with diacetyl results in the histogram shown in Figure 2c. It becomes evident that 10 residues are highly represented in the docking poses. In the following sections, we will see how these residues will be confirmed as the key players in diacetyl binding through further refinement via molecular dynamics simulations and a computational alanine scanning experiment.

We then analyzed the conformational behavior of the selected ligand. As a preliminary step, we examined all docking poses and evaluated the distribution of the distance between the two oxygen atoms in diacetyl, previously described as an indicator of the cis or trans conformation of the ligand. Figure 2d shows this distribution: the two conformations appear to be nearly equally represented, with the cis conformation (*d* ≤ 0.32 nm) accounting for approximately 40% of the poses. This contrasts with the distribution observed in aqueous solution, where the trans conformation is largely dominant. Importantly, there appears to be no correlation between the ligand conformation and the predicted binding affinity. Figure 2e displays a scatter plot, showing these two quantities for each pose. No clear trend is observed, suggesting that the ligand’s conformation has little influence on the binding affinity, at least according to the scoring function used by the docking software.

To further evaluate and gain deeper insight into the molecular details of this recognition process, we selected one docking pose with a highly favorable binding affinity and another with a highly unfavorable affinity for validation through molecular dynamics simulations. Specifically, we chose poses with a binding affinity lower than −4 kcal/mol and identified the most representative pose in terms of ligand interactions. Additionally, we selected a negative control pose by considering those with binding affinities greater than 0 kcal/mol and identifying the most representative pose within this group. The two selected docking poses are shown in Figure 2f.

### Identification of the Protein–Ligand Binding Mode

As mentioned, we proceeded to simulate both selected poses in molecular dynamics, constructing a setup equivalent to that illustrated in Figure 1, but with the addition of the ligand. The results of these simulations are illustrated in Figure 3, where the orange color represents the simulation of the pose with favorable binding affinity, while the gray color corresponds to the simulation of the unfavorable pose.

**Figure 3:**
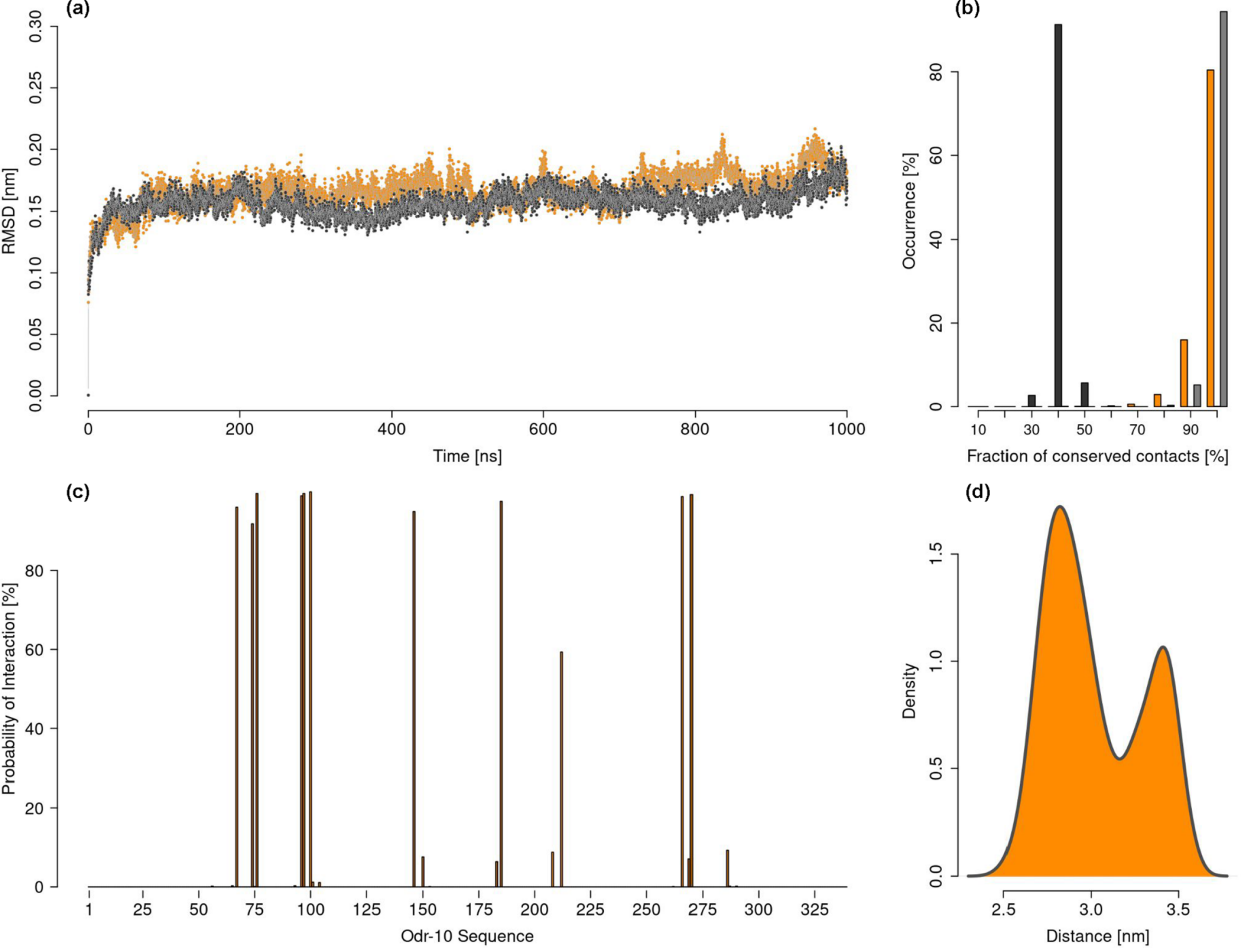
Simulation of the complex between ODR-10 and diacetyl. (a) RMSD of the simulations as a function of simulative time with respect to the initial configuration. This plot regards the transmembrane part of the peptide in the form of a plasmid of ODR-10. The orange line accounts for the pose with favorable binding affinity, while the gray line represents the pose with unfavorable binding affinity. (b) Distributions of the fraction of conserved intermolecular contacts during the simulations. Orange bars account for the contact conserved during the simulations of the favorable pose with respect to its initial configuration. Black bars account for the contact conserved during the simulations of the unfavorable pose with respect to its initial configuration. Gray bars account for the contact conserved during the simulations of the unfavorable pose with respect to the initial configuration of the favorable pose. (c) Histograms depicting the fraction of equilibrium frames, regarding the molecular dynamics of the favorable pose, in which each ODR-10 residue is in contact with the ligand. (d) Distribution of the distance values observed between the two oxygen atoms of diacetyl in all of the frames of the molecular complex simulations.

In Figure 3a, we report the RMSD relative to the initial configuration, considering only the transmembrane portion of the protein. As observed, both simulations demonstrate significant stability concerning this region of the protein, testifying to the equilibrium reached by the dynamics.

We then analyzed the protein–ligand contacts preserved throughout the simulation. Defining the initial contacts as those present in the docking pose, from which the simulation starts, we assessed the percentage of these contacts retained in each frame. This metric serves as an indicator of the quality of the docking pose. Regarding the favorable pose, the histogram in Figure 3b (orange bars) shows that, in the vast majority of frames, these contacts remain highly conserved throughout the simulation. Moreover, in all frames, we observe that at least 70% of the initial contacts are retained.

A different trend is observed for the unfavorable pose (black bars in Figure 3b). Indeed, the simulation shows a much lower contact retention, ranging between 30 and 50%, indicating that the ligand has shifted during the dynamics and that more than half of the initial contacts have been lost. This result was somewhat expected as this pose was selected among those with the most unfavorable binding affinity from an energetic perspective.

However, an even more significant aspect emerges when analyzing the percentage of contacts defined in the initial conformation of the favorable pose that are also present in the dynamics of the unfavorable pose (gray bars in Figure 3b). As clearly shown, in the vast majority of frames from the unfavorable dynamics, we observe the same contacts initially identified in the favorable pose. This finding indicates that, during the simulation of the unfavorable pose, not only did the ligand move away from its initial pocket but also it relocated to the binding site identified in the favorable pose, further supporting its validity. Notably, the distance between the centroid of the ligand in the two conformations exceeds 7 Å.

In light of these considerations, we then focused on analyzing the dynamics of the favorable pose (which, after a brief transient phase, proved to be, in many respects, equivalent to the unfavorable pose) to investigate the details of the residues most involved in diacetyl recognition. We therefore defined the interaction probability of each residue with the ligand by calculating the fraction of frames in which each residue maintains contact with diacetyl throughout the simulation. The results of this analysis are reported in Figure 3c. The prominence of certain residues, already identified in docking analysis, is evident as they represent the main molecular determinants of ligand recognition. These residues will be further examined in the next section through a computational alanine scanning experiment. The identity of the residues characterized by interaction probability higher than 90% is summarized in Table 1.

**Table 1:** Probability of Interaction with Diacetyl for the 10 Residues Most Involved in Ligand Recognition^a^

**residue**	67 MET	74 PHE	76 LEU	96 TYR	97 CYS	100 PHE	146 TRP	185 TYR	266 THR	270 PHE
**int occurrence [%]**	96.0	91.8	99.5	98.9	99.5	99.9	94.9	97.5	98.7	99.2
**hydro occ [%]**	37.7	51.7	27.1	68.3	58.6	94.6	3.7	46.5	39.0	49.0
**H-bond occ [%]**	0.0	0.0	0.0	0.0	0.0	0.0	89.3	0.1	0.0	0.0

a In the first row, we report the probability of interaction, defined based on a distance threshold between any pair of atoms. In the second row, we show the occurrence of hydrophobic contacts between each residue and the ligand, while in the last row, we report the occurrence of hydrogen bonds. All of these percentages were calculated over all frames of the molecular dynamics trajectory.

To better characterize the molecular interactions stabilizing the complex, we analyzed the nature and persistence of the contacts established between diacetyl and the residues listed in Table 1 over the MD trajectory. Two main classes of interactions were considered: hydrophobic contacts, defined as C–C interactions within 0.4 nm, and hydrogen bonds, identified using the standard gmx hbond algorithm. The average occurrence of each interaction type across the trajectory is reported in Table 1.

Overall, hydrophobic interactions are widespread among most residues in the binding pocket, with particularly high persistence for PHE100, which forms a hydrophobic contact with the ligand in approximately 80% of the analyzed frames. In contrast, TRP146 contributes primarily through hydrogen bonding rather than hydrophobic interactions: a stable H-bond (donor from the indole NH group), with one of the carbonyl oxygens of diacetyl, is observed in over 80% of the frames. These findings support the complementary roles of hydrophobic packing and specific hydrogen bonding in stabilizing the diacetyl–ODR-10 complex.

Finally, we also investigated the conformational variability of the ligand during molecular dynamics simulations of the ligand–ODR-10 complex. Specifically, we analyzed the distribution of the distance between the two oxygen atoms in diacetyl over the course of the simulation of the most favorable binding pose (Figure 3d). The results show that the cis and trans populations are again in substantial equilibrium, with a preference for the cis conformation (69% of the frames are characterized by a ligand in a cis conformation). Although this represents a marked difference from the behavior of the ligand in aqueous solution—where the trans form predominates—it suggests that the ligand can be accommodated within the binding pocket in either conformation and that its adopted configuration is not a key determinant for binding stability.

### Computational Alanine Scanning: Investigating the Role of Residues Involved in Ligand Recognition

To further analyze and confirm the role of these residues, we performed computational modeling by mutating each of these residues to alanine and studying the behavior of the mutant proteins in molecular dynamics simulations. Specifically, using PyMOL, we generated 10 mutants, each with one of the 10 residues listed in Table 1 replaced by alanine. Following the previously described simulation setup, we carried out a 100 ns long molecular dynamics simulation for each mutant. The results of these simulations are summarized in Figure 4.

**Figure 4:**
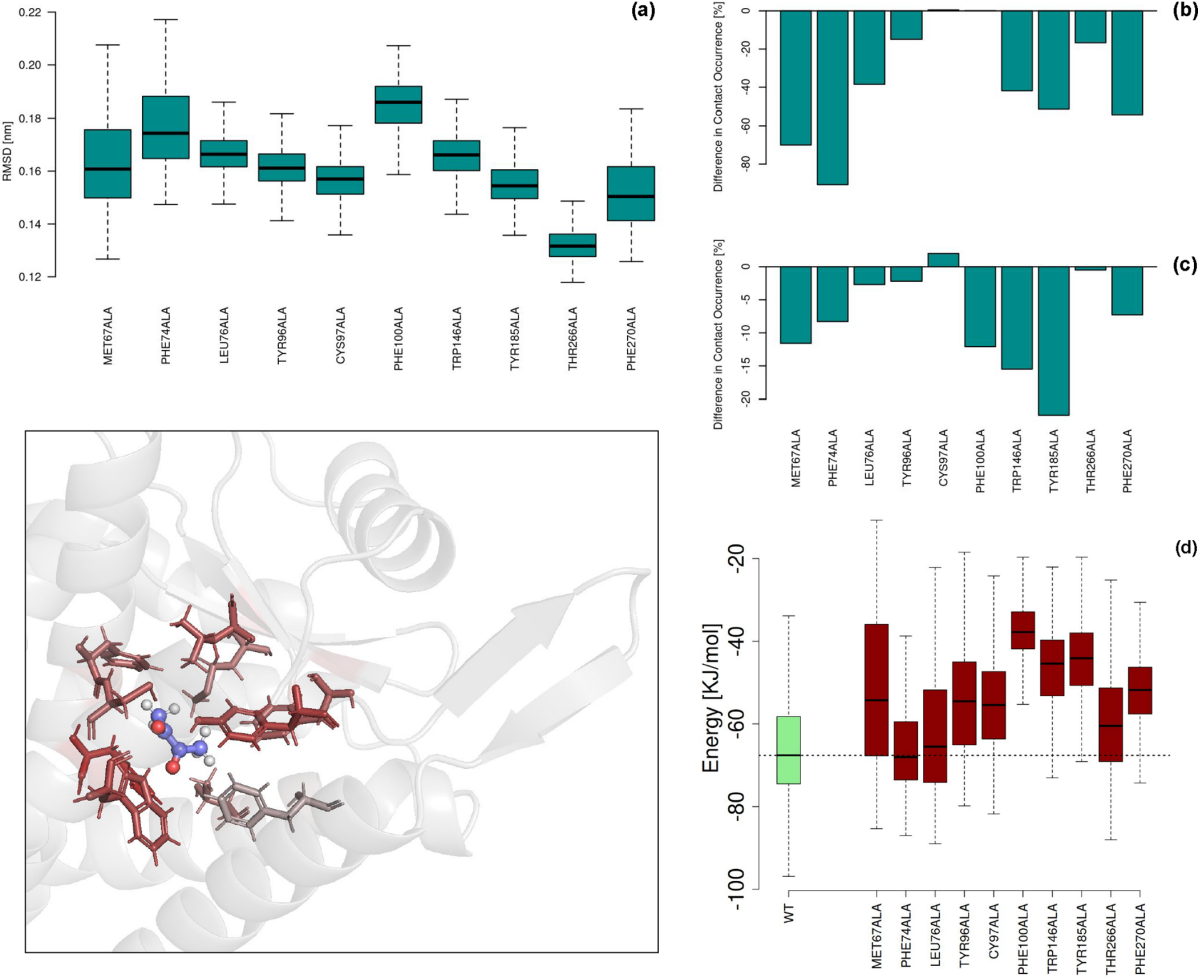
Computational alanine scanning. (a) Boxplots regarding the equilibrium RMSD for all the 10 molecular dynamics analyzed. These boxplots are focused on the transmemebrane part of ODR-10. (b) Difference in percentage between the number of contacts with the ligands observed in the WT and in the mutated simulations. This plot describes the role of each specific position. (c) Difference in percentage between the number of contacts with the ligands observed in the WT and in the mutated simulations. This plot describes the role of all 10 investigated positions. (d) Box plots represent the protein–ligand interaction energy obtained in the WT and in the mutated simulations using the MM/PBSA method. In the **left bottom** molecular representation, we report the molecular complex, where the identified 10 residues are colored according to the median of the predicted binding affinity, as a measure of their importance in binding.

First, in Figure 4a, we present a boxplot describing the equilibrium RMSD behavior (within the 25–100 ns interval) for each of the simulations, where the values refer exclusively to the transmembrane portion of ODR-10. Although the RMSD values exhibit slight variations among the different simulations, all trajectories demonstrate overall stability, remaining consistent with the values obtained in the wild-type (WT) protein simulations. This indicates that the computational modeling of each mutation did not compromise the folding of ODR-10 protein.

Next, we focused on analyzing the contacts between the protein and the ligand. Specifically, we examined the percentage variation in contacts observed in the WT simulation compared with the different mutant simulations, presenting the results in Figure 4b–c. In panel b, we present the difference in the number of contacts formed between the WT and the examined mutant, specifically analyzing the position undergoing mutation. Interestingly, we observe that in almost all cases, the substitution of the residue with alanine leads to a reduction in contacts with diacetyl. Indeed, in the most extreme cases, we observe that the substitution of phenylalanine with alanine at position 74 results in a 90% reduction in interaction with the ligand, while the substitution of methionine with alanine at position 67 leads to a 70% decrease in contacts. Similarly, it is noteworthy that mutations at positions 97 and 100 do not cause significant effects. Obviously, these effects could be due to the substitution of these residues, especially with a small and chemically inert amino acid like alanine. To overcome this limitation, we then focused on the loss of contacts involving all 10 residues defined in Table 1, reporting the effect in panel [Fig fig3]c. Significantly, in this case as well, almost all mutants exhibit a net loss of contacts, in many cases quite substantial. For example, the substitution of tyrosine at position 185 results in a reduction of over 20% of the contacts within the entire binding site, while the replacement of tryptophan at position 146 leads to a decrease of around 15%. Importantly, PHE100ALA substitution causes a relevant decrease in the overall protein–ligand contact, even if not localized in the substituted residue itself.

To integrate all this information, we estimated the binding free energy of the diacetyl–ODR-10 complex using the Molecular Mechanics Poisson–Boltzmann Surface Area (MM-PBSA) approach, as implemented in the g_mmpbsa tool.^31^ The considered contributions to the interaction energy are van der Waals, electrostatic, polar, and nonpolar terms. This analysis was performed for both the wild-type receptor and the ten alanine mutants identified through the computational alanine scanning procedure. The resulting energy distributions are shown as boxplots in Figure 4d.

The results indicate that almost all mutations lead to a reduction in binding energy compared with the wild type. Interestingly, the PHE100ALA substitution causes the most significant worsening of the interaction energy, in line with the findings from the previous section, where PHE100 was shown to be strongly involved in hydrophobic contact with the ligand. Similarly, the TRP146ALA mutation appears to be highly detrimental, likely due to the disruption of the hydrogen bond described above.

While larger differences might have been observed by accounting for cooperative effects (i.e., introducing multiple mutations simultaneously) or by extending the simulations further, both approaches would come with a substantially higher computational cost.

Considering both the intermolecular contact analysis and the calculations from MM-PBSA, we can conclude that in each case, the alanine mutation results in an overall decrease in binding, providing strong computational confirmation regarding the actual composition of the binding site.

## Conclusions

Our study provides a detailed investigation about the molecular recognition process involving ODR-10 and its agonist ligand, diacetyl, through a combination of molecular dynamics simulations and docking analyses. We found that the transmembrane portion of ODR-10, hypothesized as responsible for ligand binding, remains structurally stable over time, allowing us to sample its conformations uniformly for docking studies. The docking results highlighted ten key residues consistently involved in ligand interaction, with the most favorable binding poses showing a high degree of contact conservation during molecular dynamics simulations. Interestingly, when analyzing an unfavorable docking pose, we observed that the ligand gradually migrated toward the binding site identified in the favorable pose, reinforcing the reliability of our docking predictions. To further validate these findings, we performed a computational alanine scanning experiment, generating single-point mutants for each of the ten key residues and evaluating their impact through molecular dynamics simulations. The results consistently demonstrated a reduction in ligand interactions in most mutants, with some, such as PHE100 and TRP146, showing a particularly strong effect on binding. Moreover, it is worth noting that although significant differences are observed between the most probable conformations of the ligand in solution and when bound to the receptor, these differences do not appear to be decisive for molecular recognition.

These findings are particularly relevant within the broader context of olfactory signaling in *Caenorhabditis elegans*. The nematode’s olfactory system is highly specialized, relying on a relatively small number of sensory neurons to detect and respond to environmental cues. ODR-10 plays a central role in this process, mediating chemotaxis toward diacetyl, a key odorant for C. elegans’ food-seeking behavior. Moreover, the C. elegans olfactory system holds considerable potential for biotechnological applications. The high sensitivity and specificity of its odorant receptors make them attractive candidates for biosensor development, with possible applications in environmental monitoring, food safety, and medical diagnostics.^42,43^ Understanding the molecular determinants of ligand binding in ODR-10 not only enhances our comprehension of GPCR-mediated olfaction but also provides a framework for engineering synthetic receptors with tailored ligand specificity.

Given the central role of ORs in numerous biological processes, the ability to accurately identify key binding residues through computational methods can be helpful in bioengineering applications, from designing modified receptors with tailored ligand specificity to developing biosensors and synthetic odorant receptors.

## Materials and Methods

### Molecular Dynamics Simulation

The originally used ODR-10 structure is modeled by AlphaFold and was downloaded from Uniprot (Uniprot ID: Q18807). As is often the case for GPCR proteins, the confidence score provided by AlphaFold is very high in the extracellular and transmembrane regions, while significantly lower values are observed in the cytoplasmic regions. Nevertheless, the average per-residue confidence score (pLDDT) reaches a value of 90.3, which, according to AlphaFold’s own classification, corresponds to a “very high” level of confidence in the model. It is important to note that the simulations presented in this work were carried out without considering the palmitoylation of residue CYS317, which is often present in class A GPCRs and serves to anchor this residue to the membrane. We made this choice because this modification should not have an impact on the putative binding site, which, according to our working hypothesis, is located within the transmembrane region between the α-helices. Indeed, when comparing these results with additional simulations performed with the palmitoylated cysteine, we did not observe any significant differences (data not shown).

The diacetyl structure was downloaded from Pubchem^44^ and PubChem CID 650.

We used Gromacs 2021 to run all the all-atom classical molecular dynamics simulations.^45^ The membrane–protein–water–ion system was built using the CHARMM-GUI input generator.^46–48^ The used force field is CHARMM.^49^

When the diacetyl was alone in solution, it was placed in a dodecahedron simulative box, with periodic boundary conditions. The parametrization was built using the SwissParam web server.^50^ All molecular atoms were at least at a distance of 1.1 nm from the box borders. The minimizations were performed with the steepest descent algorithm. Next, a two-step thermalization of the system was run in NVT and NPT environments, each for 0.1 ns at a 2 fs time step. When the diacetyl is involved in molecular dynamics simulations, its topology and parameter files were generated using ParamChem.^51^

The receptor was always simulated in a heterogeneous lipid bilayer generated with CHARMM-GUI. The membrane contains the following 8 lipids, with an equimolar distribution: ERG: 12.5%; DLPA: 12.5%; DLPS: 12.5%; CER3:12.5%; DLPG: 12.5%; DMPI: 12.5%; DDPC: 12.5%; DLPE: 12.5%.

In all the simulations, a cutoff of 1.2 nm was imposed for the evaluation of short-range nonbonded interactions and the particle mesh Ewald method for the long-range electrostatic interactions.^52^ Each system was solvated with the explicit TIP3P water model into a parallelepiped simulation box.^53^ The system is neutralized using Na and Cl ions at a standard concentration.

The minimizations were performed with the steepest descent algorithm. For each simulation of membrane-embedded systems, a six-step equilibration protocol is performed according to CHARMM-GUI suggestions, followed by the production run. Using a v-rescale thermostat, the temperature was kept constant at 303.15 K. The pressure was set at 1 bar with a Parrinello–Rahman barostat.^54^ We adopted the LINCS algorithm to constrain bonds involving hydrogen atoms.^55^

All our molecular dynamics trajectories are available on Zenodo, using the following doi: 10.5281/zenodo.17409395.

### Molecular Docking

We docked diacetyl on the 42 frames, and we extracted from the molecular dynamics simulation of unbound ODR-10 using AutoDock Vina.^35,36^ In each case, the protein is rotated to have its principal axis on the *ẑ* direction, relying on the “bio3d” package in R.^56^ We restrained the possible docking poses on a 15 × 15 × 25 *Å* box. The box is constructed with the following protocol:

- Identification of the centroid of the *C*_α_-atoms of the transmembrane part of ODR-10. The residues identified as the transmembrane are, according to Uniprot definition, 12:32, 43:63, 93:113, 132:152, 202:222, 255:275, and 285:305.
- Identification of the centroid of the *C*_α_-atoms of the extracellular part of ODR-10. The residues identified as extracellular are, according to Uniprot definition, 1:11, 64:92, 153:201, and 276:284.
- Computation of the parallelepiped center as the midpoint of the line connecting the two centroids.
- Construction of the parallelepiped box spanning the 15 × 15 × 25 Å dimension in each direction around the center identified in the previous point.

This procedure overall results in 702 independent docking poses with the corresponding predicted binding affinity.
